# A novel slim cholangioscope facilitates antegrade laser lithotripsy for an intrahepatic biliary stone with acute ductal angulation

**DOI:** 10.1055/a-2707-3560

**Published:** 2025-10-02

**Authors:** Haruo Miwa, Shotaro Tsunoda, Kazuki Endo, Ritsuko Oishi, Yuichi Suzuki, Hiromi Tsuchiya, Shin Maeda

**Affiliations:** 1 26437 Gastroenterological Center, Yokohama City University Medical Center, Yokohama, Japan; 2 Department of Gastroenterology, Yokohama City University Graduate School of Medicine, Yokohama, Japan

Antegrade laser lithotripsy via endoscopic ultrasonography-guided hepaticogastrostomy (EUS-HGS) is challenging when the intrahepatic bile duct forms acute angulation 1 2 . A novel slim cholangioscope (9-Fr eyeMAX; Micro-Tech, Nanjing, China), featuring a highly flexible deflection system, has recently become available 3 4 5 .

A 78-year-old man who had undergone pancreatoduodenectomy for a neuroendocrine tumor was admitted with a large intrahepatic biliary stone ( Fig. 1 ). First, balloon enteroscopy-assisted endoscopic retrograde cholangiography was performed; however, severe adhesive angulation of the afferent limb resulted in poor maneuverability of the balloon enteroscope. Although the 9-Fr eyeMAX could be advanced with difficulty, targeting the intrahepatic stone by laser probe (LithoEVO; EDAP TMS, Lyon, France) was impossible ( Fig. 2 ). Therefore, EUS-HGS was planned to create the antegrade route for stone removal.

**Fig. 1:**
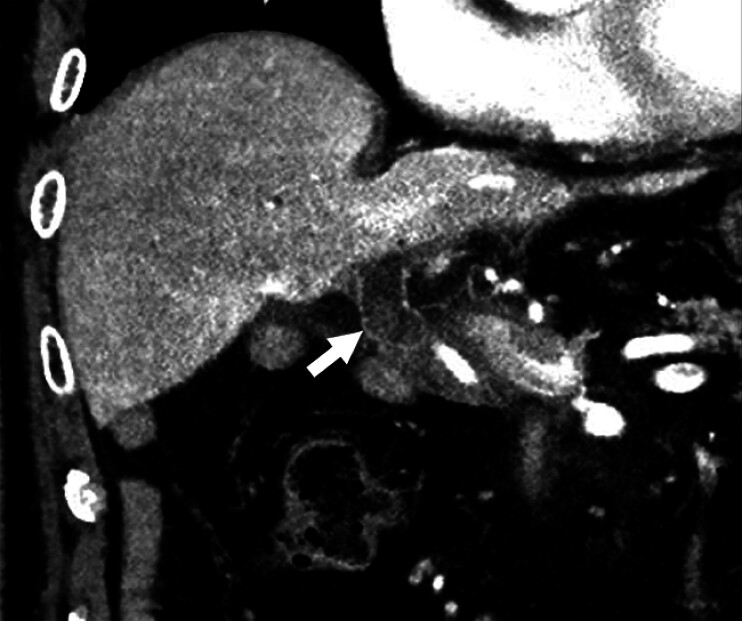
Computed tomography revealed an intrahepatic biliary stone in the coronal view (arrowhead).

**Fig. 2:**
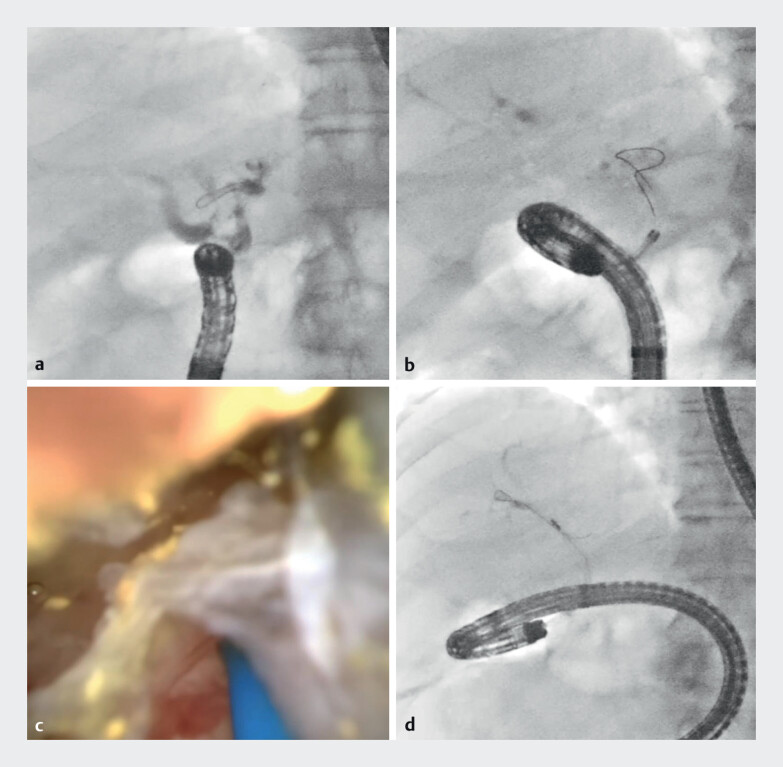
Balloon enteroscopy-assisted endoscopic retrograde cholangiopancreatography. **a** Cholangiography showed an intrahepatic biliary stone in the left hepatic duct. **b** The 9-Fr eyeMAX (Micro-Tech, Nanjing, China) advanced with difficulty into the bile duct. **c** Targeting the intrahepatic stone by laser probe was impossible. **d** A crusher basket catheter could not grasp the stone owing to acute angulation.

A non-dilated bile duct, identified posterior to a hepatic cyst, was punctured with 22-gauge needle. After guidewire placement toward the hilum, a double-pigtail plastic stent was deployed ( Fig. 3 ).

**Fig. 3:**
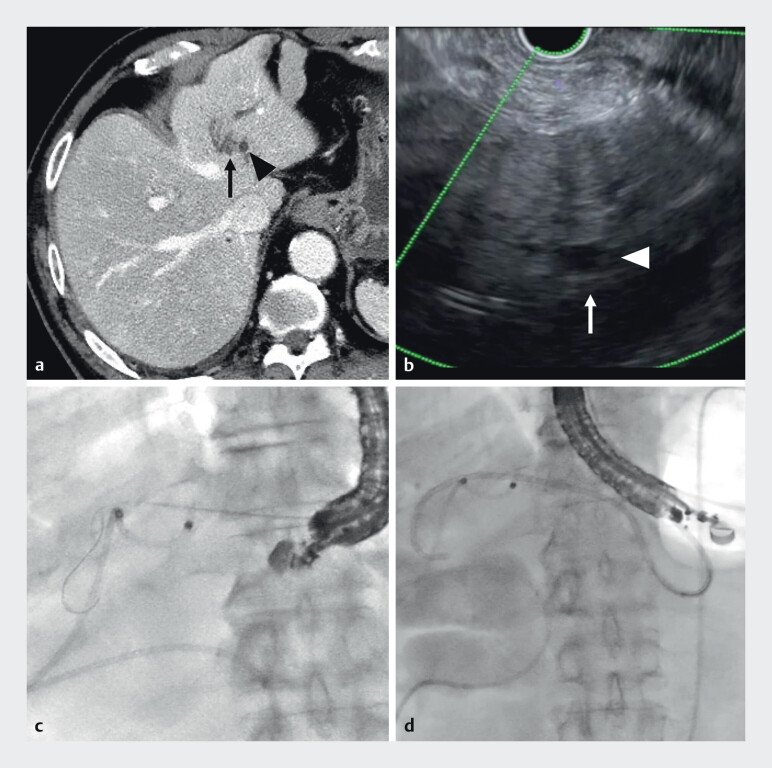
Endoscopic ultrasonography-guided hepaticogastrostomy. **a** Computed tomography showed the intrahepatic bile duct (arrow) and a hepatic cyst (arrowhead). **b** Endoscopic ultrasonography showed a non-dilated bile duct (arrow), located posteriorly to a hepatic cyst (arrowhead). **c** The intrahepatic bile duct was punctured with a 22-gauge needle, and a guidewire was inserted toward the hilum. **d** A double-pigtail plastic stent was deployed.

Three weeks later, the plastic stent was exchanged for a partially covered metallic stent, and antegrade stone removal was performed. The 9-Fr eyeMAX was inserted through the metallic stent; however, targeting the intrahepatic stone was difficult because of the acute intraductal angulation. Rotation of the cholangioscope and bile aspiration brought the stone into direct frontal view. Although the laser probe tip intermittently went out of view, the green aiming beam allowed clear discrimination between the stone and the bile duct wall. After laser lithotripsy, the hepaticojejunostomy anastomosis was dilated with a balloon catheter, and the fragmented stones were successfully removed. No residual stones were seen on cholangioscopy, and the metallic stent was removed ( Fig. 4 ). The patient was discharged without complications.

**Fig. 4:**
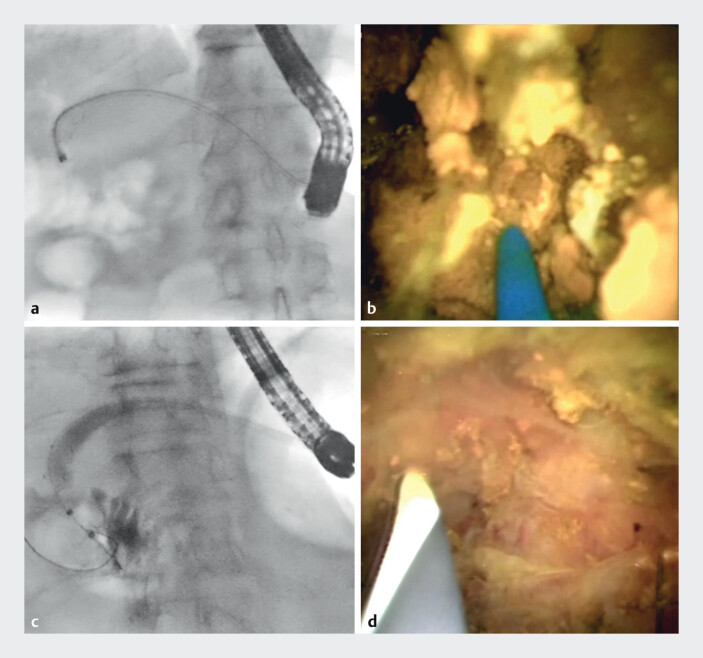
Peroral cholangioscopy-guided laser lithotripsy. **a** The 9-Fr eyeMAX (Micro-Tech, Nanjing, China) was inserted through the metallic stent. The intrahepatic bile duct showed acute angulation. **b** Laser lithotripsy was successfully performed. **c** The fragmented stones were removed to the afferent limb. **d** Cholangioscopy revealed no residual stones.

To the best of our knowledge, this is the first report of antegrade laser lithotripsy for intrahepatic biliary stones using a novel slim cholangioscope ( Video 1 ).

A novel slim cholangioscope enabled antegrade laser lithotripsy for an intrahepatic biliary stone with acute angulation of the bile duct.Video 1

Endoscopy_UCTN_Code_TTT_1AR_2AH

## Notes

Endoscopy E-Videos https://eref.thieme.de/e-videos
*E-Videos* is an open access online section of the journal *Endoscopy* , reporting on interesting cases and new techniques in gastroenterological endoscopy. All papers include a high-quality video and are published with a Creative Commons CC-BY license. Endoscopy E-Videos qualify for HINARI discounts and waivers and eligibility is automatically checked during the submission process. We grant 100% waivers to articles whose corresponding authors are based in Group A countries and 50% waivers to those who are based in Group B countries as classified by Research4Life (see: https://www.research4life.org/access/eligibility/ ). This section has its own submission website at https://mc.manuscriptcentral.com/e-videos .
